# EEG-Derived Functional Connectivity Patterns Associated with Mild Cognitive Impairment in Parkinson’s Disease

**DOI:** 10.3390/bs11030040

**Published:** 2021-03-23

**Authors:** Alejandro Armando Peláez Suárez, Sheila Berrillo Batista, Ivonne Pedroso Ibáñez, Enrique Casabona Fernández, Marinet Fuentes Campos, Lilia Morales Chacón

**Affiliations:** 1 Movement Disorders and Neurodegeneration Clinic, International Center for Neurological Restoration, Playa, Havana 11300, Cuba; ivon@neuro.ciren.cu (I.P.I.); ecasabona@neuro.ciren.cu (E.C.F.); 2 Department of Clinical Neurophysiology, International Center for Neurological Restoration, Playa, Havana 11300, Cuba; sheila@neuro.ciren.cu; 3 Polyclinic “28 de Enero”, Playa, Havana 11300, Cuba; marinetfuentes@gmail.com

**Keywords:** Parkinson’s disease, mild cognitive impairment, electroencephalogram, functional connectivity, graph theory

## Abstract

Objective: To evaluate EEG-derived functional connectivity (FC) patterns associated with mild cognitive impairment (MCI) in Parkinson’s disease (PD). METHODS: A sample of 15 patients without cognitive impairment (PD-WCI), 15 with MCI (PD-MCI), and 26 healthy subjects were studied. The EEG was performed in the waking functional state with eyes closed, for the functional analysis it was used the synchronization likelihood (SL) and graph theory (GT). RESULTS: PD-MCI patients showed decreased FC in frequencies alpha, in posterior regions, and delta with a generalized distribution. Patients, compared to the healthy people, presented a decrease in segregation (lower clustering coefficient in alpha *p* = 0.003 in PD-MCI patients) and increased integration (shorter mean path length in delta *(p* = 0.004) and theta *(p* = 0.002) in PD-MCI patients). There were no significant differences in the network topology between the parkinsonian groups. In PD-MCI patients, executive dysfunction correlated positively with global connectivity in beta (r = 0.47) and negatively with the mean path length at beta (r = −0.45); alterations in working memory were negatively correlated with the mean path length at beta r = −0.45. CONCLUSIONS: PD patients present alterations in the FC in all frequencies, those with MCI show less connectivity in the alpha and delta frequencies. The neural networks of the patients show a random topology, with a similar organization between patients with and without MCI. In PD-MCI patients, alterations in executive function and working memory are related to beta integration.

## 1. Introduction

Parkinson’s disease (PD) is a synucleinopathy with pathogenic mechanisms that produce a severe and progressive dysfunction of the connectivity between the gray nuclei of the base and the cortex, with alteration of multiple neurotransmitters [1], which explains the broad spectrum of symptoms that includes cognitive dysfunctions [2].

According to the severity of cognitive symptoms in PD, three different stages can be distinguished: patients without cognitive impairment (PD-WCI), with mild cognitive impairment (PD-MCI), and with dementia (PD-D) [2]. MCI is evident in 15–20% of de novo untreated patients, with estimates of 20–60% of patients showing some abnormality after diagnosis [3]. In addition, it is considered a risk factor for the development of dementia [3], which is associated with poorer quality of life, risk of institutionalization and increased mortality of patients, greater stress on the caregiver, and serious socioeconomic repercussions [3]. Showing the importance of studying this initial stage, for which there are still no validated markers.

Neuroscience considers the human brain as a network with anatomical (neural synapses) and functional (synchronization of neural activity) connections [4], from these, the cognitive functions would emerge as a result of the global integration of the specialized brain regions at the local level [5]. The analysis of these single functional connections through the study of functional connectivity (FC), defined as the temporal dependence of neuronal activity between two brain regions anatomically separated or not; and the study of the whole network through graph theory (TG) [4,6] it would be useful as a probable marker to evaluate MCI.

For this functional study, the electroencephalogram (EEG) has the advantage of great temporal resolution to study non-invasively the oscillations of the cerebral networks at the cortical level [5,7]. From a theoretical perspective, it is a functional marker of neuronal and synaptic integrity, which may be sensitive to subtle changes that precede the structural alterations of neurodegenerative diseases such as PD [8].

Several authors have studied the usefulness of EEG-derived measurements of FC and GT to assess cognitive manifestation in PD, although most focus on the dementia stage. These reports are consistent with a decrease in CF, as well as segregation and functional integration of the network, mainly in the alpha band [5,9,10,11,12]. Understanding the functioning of the neural connection in the stage in which the cognitive alterations begin is important for the early diagnosis, an accurate prognosis, and effective treatment of cognitive damage, which allows one to reverse or slow down its progress towards dementia. However, just a few investigations have evaluated the patterns of FC derived from EEG in PD-MCI patients [12,13] and only one the topology of the networks [12]. They reported alterations in connectivity in these patients, although with different results among the studies. Therefore, the objective of this investigation was to evaluate which EEG-derived FC patterns are associated with mild cognitive impairment in PD.

## 2. Materials and Methods

### 2.1. The Universe and Sample

A cross-sectional analytical study was carried out during the period from January 2018 to January 2020. Among patients with PD, according to the criteria of the London Brain Bank [14], attended in the consultation of Movement Disorders Clinic of the International Center for Neurological Restoration (CIREN), were included 30 that met the inclusion and exclusion criteria. The patients were divided into two groups: PD-WCI and PD-MCI, following the criteria diagnostics of the MCI proposed by the MDS [15]. Also were randomly selected 30 healthy subjects according to their age and sex compatibility with the patients to avoid the potential changes in functional connectivity related to aging. They served as a reliable comparison framework to assess whether the patterns of functional connectivity were close to or far from normal values since these are not standardized.

Inclusion criteria:

1. Patients with Parkinson’s disease, according to the diagnostic criteria of the London Brain Bank [14].
2. Patients with a complete neuropsychological evaluation, including Mattis Scale for Dementia Assessment-2 (MDRS-2), suggested by MDS [15] to determine cognitive phenotypes: PD-MCI with a score between 123–138 and PD-WCI greater or equal to 138.
3. Patients with an EEG in a waking functional state with eyes closed.

Exclusion criteria:

1. Subjects treated with drugs that can contaminate the brain electrical activity recorded in the EEG, such as anxiolytics or antipsychotics.
2. Parkinson’s disease patients with dementia according to MDS criteria [16].
3. Subjects presenting an EEG with multiple artifacts do not allow obtaining an adequate number of windows for spectral analysis.

### 2.2. Obtaining Information

The information was obtained directly from the clinical and electrophysiological evaluation of patients in the best “ON” state with respect to dopaminergic medication. These evaluations were carried out by more than one evaluator, including specialists in Neurology, Neurophysiology, and Neuropsychology. The cognitive and motor examination was performed the day before the EEG. PD medication was converted to levodopa equivalents as per previous reports [12]. The general data of the subjects and the results of the evaluations were stored in a database in Microsoft Excel 2016.

For the motor evaluation, the following were applied:

- Section III of the Movement Disorder Society-Unified Parkinson’s Disease Rating Scale revised by the Unified Parkinson’s Disease Rating Scale (MDS-UPDRS) (Annex 2).

In addition, a battery of neuropsychological scales, validated and recommended by the MDS, was applied to all patients [15] for use in the PD-MCI:

- Global Cognition: assessed by MDRS-2 a multidimensional battery to assess cognitive engagement, consists of 36 tasks divided into five subscales: attention, initiation/perseverance, construction, conceptualization, and memory. The total score, between 0 and 144, is derived from the sum of the partial scores. The MDS suggests the use of MDRS-2 with a cut-off point of ≤123 for PD-D [17] and a cutoff ≤ 137 for PD-MCI [18].
- Executive Functions: Frontal Function Assessment Test (FAB) Frontal Assessment Battery) which consists of 6 subtests that explore each of the processes controlled by the frontal lobes. Higher test scores mean better performance and the maximum total score that can be obtained on the FAB is 18 [19].
- Working memory—Attention: Evaluated by the subscale for ordering numbers and letters, part of the Wechsler Adult Intelligence Scale III (WAIS III, from the English Wechsler Adult Intelligence Scale III). It is considered adequate to be applied in patients with PD according to the MDS [15], evaluates the number of numbers and letters that can be evoked, the more combinations the better the patient.

All participants underwent an EEG in a waking functional state, with the scalp free of fat and impurities (after washing it), without the application of gel, creams, or oils that could increase the resistance of the skin to contact with the electrodes. The impedance was kept below 5 KOhm. The electrodes were placed on the scalp according to the international 10/20 system, with reference placed at the level of the ears. The duration of the test was approximately 30 min at a sampling frequency of 200 Hz, using the MEDICAID V Amplifier System Neuronic Cuba equipment. Filtering was performed with a 0.5–30 Hz bandpass (12 dB/oct.).

For the quantitative analysis of the EEG, 45 artifact-free windows were selected from the records. The frequency bands were taken in the ranges: alpha 8–12 Hz; beta 13–20 Hz; theta 4–7 Hz; delta 1–4 Hz.

The functional connectivity variable evaluated was the synchronization likelihood probability (SL) between the electrodes, as well as the properties of the network derived from it, using programming implemented in MatlabR2014a.

- SL: In all the selected segments, the spatial synchronization matrix between the electrodes was calculated for the alpha, beta, theta, and delta frequency bands. This index is based on quantifying the probability that a segment of a Y1 signal resembles another segment of the same Y2 signal, each time that in the other series X1 resembles X2. Its range is between 0 ≤ SL ≤ 1 [4,20].

The topological analysis of the network, based on graph theory, was carried out from the SL of all possible combinations of electrodes. In this network, the electrodes represent the nodes and the edges represent the functional connections established by the SL. For the topological quantification, the parameters evaluated were:

- Functional segregation measures: clustering or segregation coefficient (proportion of connections between the closest nodes, relative to the maximum number of possible connections) and local efficiency (it reflects how connected neighboring nodes are).
- Measures of functional integration: mean path length (minimum number of edges that must pass from one node to another; it reflects the efficiency of communication in a network) and global connectivity (global connectivity of each node with the rest).

### 2.3. Statistical Analysis of the Information

For statistical processing, the program Statistica 8.0 (Copyright StatSoft.Inc) and the program Matlab R2014a was used. In the clinical and demographic characterization of the sample, measures of central tendency were used, calculating the mean and standard deviation. The difference between the groups was established by the Mann–Whitney U test, with a significance value of *p* < 0.05.

To identify EEG-derived FC patterns and established the differences between the synchronization matrices of each group evaluated, was used a tool implemented in Matlab R2014a developed at the Center for Neuroscience of Cuba [21] that employed a permutations test that uses a student’s t as with *p* < 0.05. Network topology measurements were processed using the Statistica 8.0 program, for the differences between the groups, a Mann-Whitney U was used, with a significance value of *p* < 0.05. The results were presented in box and whisker graphs were generated in the same program. The relationship between topological properties of neural networks and cognitive manifestations in PD-MCI patients was performed using a spearman’s correlation test, the significance value was established as *p* < 0.05.

### 2.4. Ethical Aspects

This project was previously approved by CIREN Ethics Committee in Clinical Research in 2017 (number CEI012017).

The patients were explained about their clinical situation and the reasons why they were proposed to be included in the research. All the people who participated were informed of the study objectives and their potential benefits, using practical and understandable language. It was explained to them that the information related to the identity of the patient would be treated confidentially and that this data could only be reviewed by the personnel in charge of the investigation. Those who agreed to participate in the research after reading and signing the informed consent form (Annex 6). The principles governing medical ethics were taken into account at the time of each of the evaluations.

## 3. Results

Table 1 shows the 30 PD patients without dementia studied and their distribution into two groups: 15 with PD-MCI and 15 PD-WCI, according to the score on the MDRS-2. It is observed that the PD-MCI patients present higher motor MDS-UPDRS values, levodopa equivalent doses, average age, years of evolution, and lower FAB, WAIS-III (*p* = 0.04), and MDRS-2 (*p* = 0.00) scores than normal cognitive. Although the differences were significant only in the last two. Both groups have the same sex distribution.

### 3.1. Functional Connectivity Analysis

**Figure 1:**
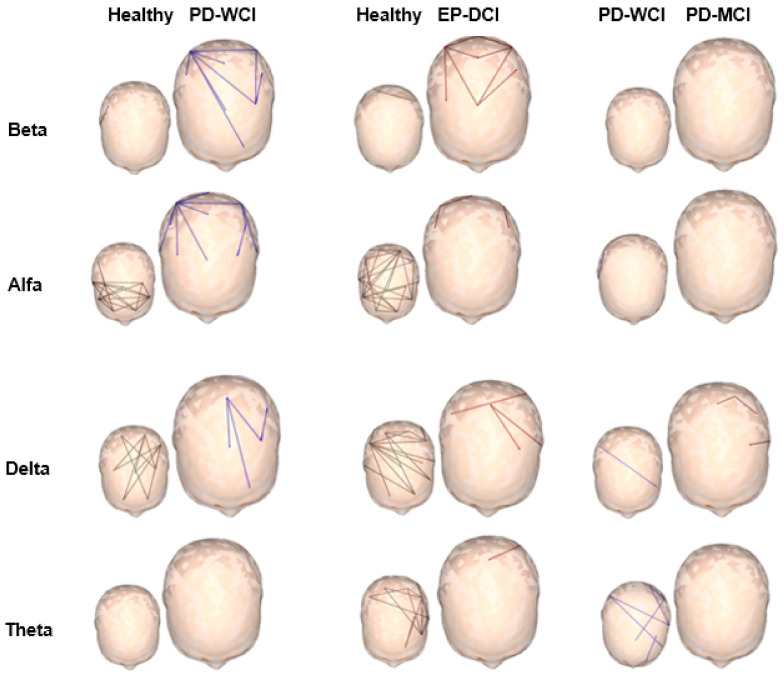
Significant connections between electrode pairs for each EEG frequency (alpha, beta, theta, and delta). The different colors represent the significance values *p* < 0.05 of the permutations test used for each of the groups studied: healthy subjects in black, patients without cognitive impairment (PD-WCI) in blue, and patients with mild cognitive impairment (PD-MCI) in red. In the skull-shaped figures, the largest size does not represent statistical differences, it is used to highlight the result of the group of greatest interest according to the objectives of the investigation.

Figure 1 presents the analysis of the FC patterns using the SL method, showing the connections between pairs of electrodes with significant differences between the studied groups: healthy subjects, PD-WCI and PD-MCI. This comparison was made with a permutations test that uses a student’s T with a significance level of *p* < 0.05, the following results were obtained:

Compared with healthy people, both groups of patients showed a decrease in synchronization in the alpha and theta frequencies, as well as an increase in the beta band in posterior regions. The reduction of the synchronization in the alpha frequency, shows spatial differences, decreases in anterior regions in the two groups of patients, while in those with PD-MCI it reaches the posterior regions. Patients with cognitive alterations were the only ones to have changes in connectivity in the delta band, showing a reduction compared to healthy subjects.

In the comparison between parkinsonian groups, changes in functional connectivity occurred in the alpha and delta bands. The PD-MCI group with respect to the PD-WCI group showed less synchronization in the alpha frequency in few connections in posterior regions and the delta band in multiple connections with a generalized distribution. There were no changes in the beta band, and the behavior in the theta frequency was very similar between both groups.

In a general sense, the two groups of PD patients presented an increase in connectivity in the beta frequency and a decrease in the theta band, regardless of cognitive alterations. Patients with PD-MCI presented lower connectivity for the alpha and delta bands with posterior and generalized topography, respectively.

### 3.2. Topological Analysis of Neural Networks

**Figure 2:**
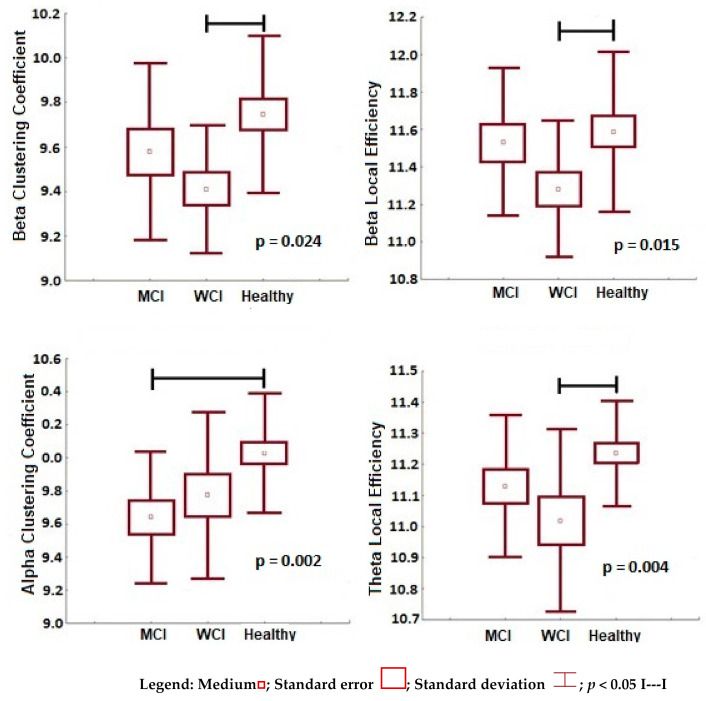
Functional segregation variables (beta clustering coefficient, beta local efficiency, alpha clustering coefficient, theta local efficiency) with significant differences between the groups (healthy subjects, PD-WCI, PD-MCI). Mann–Whitney U, significance value *p* < 0.05.

**Figure 3:**
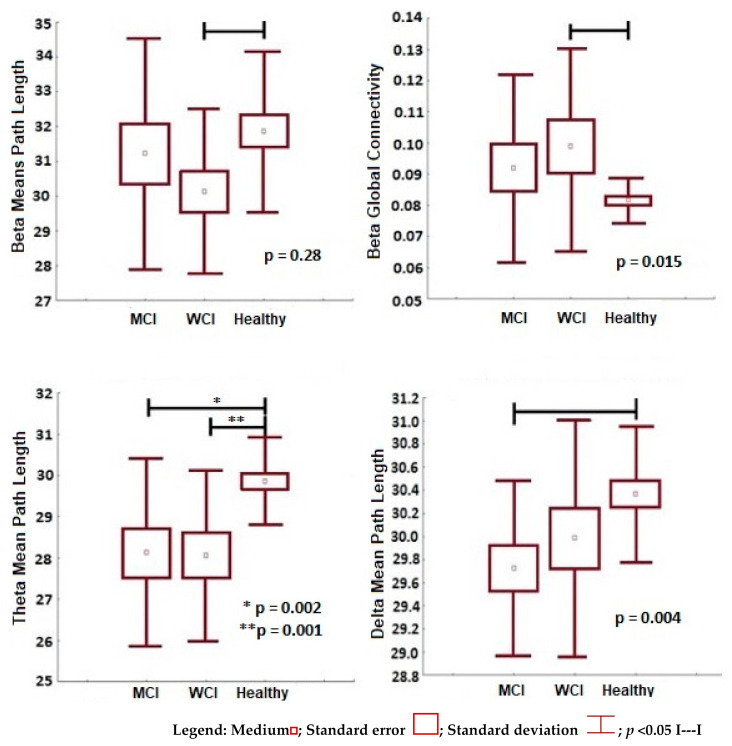
Functional integration variables (beta mean path length, beta global connectivity, theta mean path length, delta mean path length) with significant differences between the groups (healthy subjects, PD-WCI, PD-MCI). Mann–Whitney U, significance value *p* < 0.05.

The significant results of the analysis by frequencies of the topology measures of neural networks, segregation, and integration, are presented in Figure 2 and Figure 3 respectively.

Figure 2 shows the functional segregation, a decrease in these measures is observed in the two parkinsonian groups with respect to healthy subjects, but no significant differences were found between them. With healthy people as a reference, PD-WCI patients presented a reduction in functional segregation in the beta bands (lower local efficiency *p* = 0.003 and lower clustering coefficient *p* = 0.024) and theta (lower clustering coefficient *p* = 0.004); while those with PD-MCI showed this pattern only in the alpha frequency (lower clustering coefficient *p* = 0.003). It is interesting that with PD-MCI subtype maintained the trend of lower segregation (lower clustering coefficient), compared to the PD-WCI group, only in the alpha frequency, despite the were no statistical differences (*p* < 0.05) between both subtypes.

The frequency analysis of the functional integration significant measures (*p* < 0.05) between the study groups, is presented in Figure 3. In general, an increase in the integration measures is shown in the two groups of patients compared to healthy subjects, with no significant differences between parkinsonian groups.

Taking healthy people as a reference, PD-WCI patients presented an increase in functional integration in the beta frequencies (shorter mean path length *p* = 0.028 and greater global connectivity *p* = 0.015) and theta (lower mean path length *p* = 0.002). While PD-MCI patients presented an increase in integration in theta frequency (shorter mean path length), and also in the delta band (shorter mean path length *p* = 0.002). In this last frequency, it was the only one in which the trend of less integration was maintained in patients with PD-MCI compared to PD-WCI patients, despite the fact that there were no statistical differences (*p* < 0.05) between both groups.

In summary, both groups of patients presented networks with similar properties, less segregation, and greater integration, which corresponds to a random topology. The patients with PD-MCI only presented this pattern with respect to the two groups in frequencies: alpha, with decreased segregation, and delta, with increased integration; although with significant differences only compared to healthy subjects.

### 3.3. Topological Measures and Cognitive Manifestation in PD-MCI Patients

**Figure 4:**
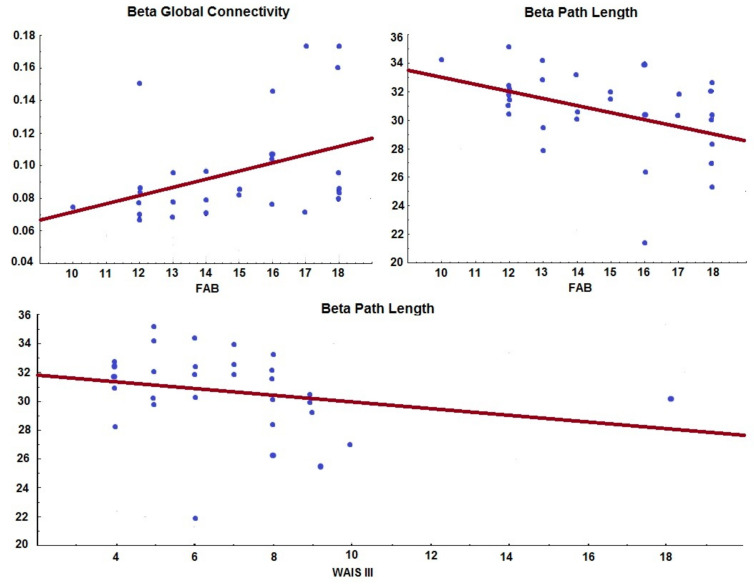
Relationship between the cognitive performance assessed by the FAB (executive functions) and WAIS-III (working memory) scales and the functional integration variables of the network (beta mean path length and beta global connectivity). Spearman correlation (*p* < 0.05).

In patients with PD-MCI, the relationship between cognitive manifestations, evaluated by neuropsychological tests (MDRS-2, FAB, and WAIS-III), and the topological properties of networks: segregation (clustering coefficient and local efficiency) and integration (means path length and global connectivity). Figure 4 shows the results of a Spearman correlation with a significant value of *p* < 0.05. Alterations in executive functions and working memory were associated with less functional integration in the beta frequency.

Executive dysfunction, expressed by a lower score in the FAB test, correlated with lower global integration in the beta band (lower global connectivity r = 0.47 and longer median path length r = −0.45). While low scores in the WAIS-III test, worse functioning in working memory, also correlated with less integration in the beta band (greater length of the mean path r = −0.45).

## 4. Discussion

The study of functional connectivity patterns in patients with PD-MCI is clinical and investigative important to clarify the pathophysiological mechanisms that explain these alterations and their progression to dementia.

In our research, two groups of patients with different cognitive statuses and without significant differences in terms of motor impairment, average age, and years of disease evolution were studied. In the PD-MCI group, there is significantly greater involvement in the working memory domain, one of the first to be affected at this stage [2,15,22,23]. They also showed involvement in executive functions, the other domain that is generally affected in this stage [2,15,22,23], although without significant difference between the groups. Therefore, we are facing a sample of patients with very similar clinical characteristics, in which cognitive decline is beginning in those with PD-MCI.

In patients with cognitive alterations, the results show a decrease in FC in the alpha frequency in posterior regions and in the delta band with a more generalized topography in relation to PD-WCI patients.

Among the reports that used frequency analysis of the FC in PD, derived from EEG or MEG, there is consensus to consider an association between the decrease of these patterns in the alpha band and cognitive impairment, although with topographic differences [10,12,24,25,26,27,28]. A loss of connectivity in this frequency has been reported in frontotemporal regions, in non-demented patients with executive dysfunction [27], and patients with non-specified cognitive alterations [24], although the MCI criterion proposed by the MDS was not applied [15].

Babiloni et al. reported disconnection in the alpha band in posterior regions in patients with MCI, due to PD or Alzheimer’s disease (AD), compared with healthy subjects, although with no difference between the groups of patients [13]. These same authors evaluated patients with dementia caused by PD or AD, reporting the same pattern [29]. With these results, they suggested that a decrease in connectivity in the alpha frequency may be related to ascending cholinergic systems, based on the fact that these pathways are altered in both pathologies and on the effect that anticholinergic drugs produce on the alpha frequency [13]. This is an interesting hypothesis that must be contrasted with studies designed for this purpose.

On the other hand, in patients with PD-D, the loss of connectivity in the alpha band in anterior and posterior regions is also the most replicated finding in the literature [10,12,24,25,26,27,28]. It has been suggested that these changes in FC during PD, with some spatial specificity in relation to cognitive decline, would have a consistent relationship with the progression of the disease [5]. In summary, our results support the evidence that indicates that functional disconnection in the alpha frequency is associated with cognitive impairment in this pathology.

The other pattern that appeared in patients with MCI was a decrease in synchronization in the delta band. However, there are contradictory results between the studies FC, derived from EEG or MEG, in the delta frequency in PD patients with cognitive alterations. While some authors report an increase in connectivity [12,13], others, like our finding, report a decrease in this frequency [10,25,28]. There are multiple differences between these reports in the methods to assess FC and in the scales that define cognitive impairment, which may partly explain these opposite results.

The two groups of patients with PD presented an increase in synchronization in the beta frequency and a decrease in the theta band compared to healthy subjects; between them, the FC patterns were very similar regardless of the presence of cognitive impairment. Several authors have reported an association between the increase in CF in the beta band and the motor impairment of PD, which is restored with dopaminergic therapy [30,31,32]. In the theta band, contradictory results have been reported, some have found an increase in connectivity in patients without cognitive alterations [33], with MCI [12], and with dementia [26]. While our result agrees with Basboom et al. who reported a decrease in PD-D patients [10]. Although there are differences in the methodology in these studies, they all point to the fact that FC is altered at these frequencies in PD patients, although it is not clear that they are related to cognitive impairment, a fact that is also manifested in our results. Further studies with large groups of patients must be carried out to clarify these patterns.

In summary, our results agree with the studies that suggest that the connectivity of the oscillatory activity at cortical level is altered in all frequencies in patients with PD, even in the initial stages, when it is assumed that the neo-cortex would not present pathological lesion according to Braak stadiums [34,35]. These changes in the alpha and delta frequencies may be related to mild cognitive impairment in this pathology.

After studying the synchronization of functional connections, the organization of the entire network was analyzed in the PD patients. An efficient network in a healthy person works theoretically according to the small world model, in which local segregation and global integration are balanced, combining a high density of neural connections at the local level with the existence of some long-range connections [4]. In our study, both groups of patients presented networks with an architecture that deviates from this model and shows a random topology compared to healthy subjects. These networks were characterized by a decrease in segregation and an increase in global integration, with some differences by frequencies in the PD-MCI group.

The functional segregation in patients with cognitive impairment showed a decrease in the alpha band. A progressive loss of functional segregation in this frequency occurred in patients with de novo PD without cognitive alterations, who were followed for four years, in a MEG-based study [36]. This pattern has also been reported in studies derived from EEG, in relation to MCI and dementia caused by PD [12], as well as in patients with AD [37]. These findings imply less information exchange at the local level, and therefore, randomization of neural networks. These alterations occur in the alpha frequency, which is normal in an awake state with eyes closed.

Regarding the integration measures in PD-MCI patients compared to healthy subjects, there was an increase for the slow bands, delta, and theta, although with no changes between the parkinsonian groups. Functional integration is a measure of information transmission from one part of the network to a distant one, therefore, an increase is occurring in these exchanges in these frequencies, pathologically in healthypeople in the functional state evaluated.

In the only EEG-derived graph theory study conducted in PD-MCI patients so far, there were no changes in functional integration measures compared with patients without cognitive alterations. However, PD-D did show an increase in integration compared to those with PD-WCI, although in the alpha band [12]. In Alzheimer’s patients, an increase in integration in the theta band has also been reported, a pattern similar to presented in our study [38].

The neurophysiological mechanism that generates these slow waking frequencies is poorly understood [29]. Although, if it is known that the anatomical or functional disconnection between related cortical areas, generates spontaneous slow oscillations in practically all the registered neurons. Therefore, the increase in global integration at these frequencies can be seen as the effect of the disease on neural networks [38].

In general, both groups of patients presented random networks, with similar properties, which may be due to the few clinical differences between the PD patients in the study. Although, it is noteworthy, that patients with PD-MCI showed this random architecture in the alpha and delta frequencies, the same in which they presented a decrease in CF.

When relating the topological measures of the networks of PD-MCI patients and the cognitive manifestations evaluated, we found that a decrease in integration in the beta frequency was related to greater alterations in executive functions and working memory.

This result has not been replicated in other investigations so far. In patients with PD, a relationship between the loss of segregation in the alpha frequency and global cognition evaluated by the mini-mental state exam (MMSE) is reported [12]; however, the MDS does not advise this scale in patients with PD to assess MCI [15], because does not evaluate executive functions, precisely one of the domains in which we obtained our results. Another study linked executive dysfunction with loss of coherence in the alpha frequency [27], a CF pattern and not network theory, also with a different methodology than the one we use. In patients with AD, topological changes in the beta band were associated with lower scores on the MMSE [37], but in measures of segregation and not of integration, as was found in our study. Due to the few investigations of neural networks derived from the oscillatory brain activity in PD patients with cognitive alterations, the description of our results reaches greater importance, being able to contrast with future investigations.

In general, we can state that patients with PD-MCI presented a decrease in functional connectivity and random neural networks at the cortical level. Several authors have reported these and greater alterations in functional connectivity in the dementia stage, defining it as a syndrome of functional disconnection [12,13,28,39]. Our investigation shows that some of these alterations are already occurring in the previous stage of cognitive decline. Therefore, the study of EEG-derived functional connectivity patterns in PD patients can be a useful tool to assess MCI.

## 5. Conclusions

Parkinson’s disease patients present alterations in functional connectivity in all frequencies, those with mild cognitive impairment show less functional connectivity in alpha and delta bands.

Neural networks in Parkinson’s disease show a random topology, characterized by a loss of segregation and an increase in integration, with similar functioning between patients with and without mild cognitive impairment.

In Parkinson’s disease patients with mild cognitive impairment, the alterations in executive function and working memory are related to loss of global integration in the beta frequency.

## Figures and Tables

**Table 1:** Clinical and demographic characteristics of patients with Parkinson’s disease.

	PD-WCI *n* = 15 M (SD)	PD-MCI *n* = 15 M (SD)	PD Total M (SD)	*p*
**Average age**	58.40 (7.4)	62.73 (7.8)	60.57 (7.8)	0.13
**Sex (M/F)**	4/1	4/1	4/1	-
**Years of Evolution**	7.73 (4.5)	8.06 (4.5)	7.90 (4.4)	0.84
**FAB**	15.13 (2.5)	14.33 (2.4)	14.73 (2.5)	0.08
**MDRS-2**	140.67 (1.7)	130.93 (5.04)	135.80 (6.2)	0.00
**WAIS III**	7.93 (3.3)	5.93 (1.6)	6.93 (2.8)	0.04
**MDS-UPDRS Motor**	31.73 (9.6)	36.00 (12.32)	33.86 (11.1)	0.34
**LED**	568.3 (201.4)	588.3 (343.4)	578.3 (276.8)	0.07

M: mean, SD: standard deviation, M/F: male to female ratio, FAB: Frontal Function Assessment Test, MDRS-2: Mattis Scale for Dementia Assessment-2, WAIS-III: Wechsler Adult Intelligence Scale III, MDS-UPDRS: Movement Disorder Society-Unified Parkinson’s Disease Rating Scale revised by the Unified Parkinson’s Disease Rating Scale; LED: levodopa equivalent dose.

## Data Availability

Not applicable.
